# Supplementary material for: Glycolytic reprograming in Salmonella counters NOX2-mediated dissipation of ΔpH
Source: Nat Commun. 2020 Apr 14;11:1783. doi: 10.1038/s41467-020-15604-2 (PMC7156505; doi:10.1038/s41467-020-15604-2)
Supplement: Supplementary file 1 — Supplementary Information [file 41467_2020_15604_MOESM1_ESM.pdf]

Supplementary Information

Glycolytic reprograming in *Salmonella* counters NOX2-mediated dissipation of ΔpH

Sangeeta Chakraborty[1], Lin Liu[1], Liam Fitzsimmons[1], Steffen Porwollik[2], Ju-Sim Kim[1], Prerak Desai[2], Michael McClelland[2], Andres Vazquez-Torres[1,3]*

[1]Department of Immunology & Microbiology, University of Colorado School of Medicine, Aurora, CO
[2]Department of Microbiology and Molecular Genetics, University of California Irvine School of Medicine, Irvine, CA
[3]Veterans Affairs Eastern Colorado Health Care System, Denver, CO

*Correspondence:
E-mail: andres.vazquez-torres@cuanschutz.edu

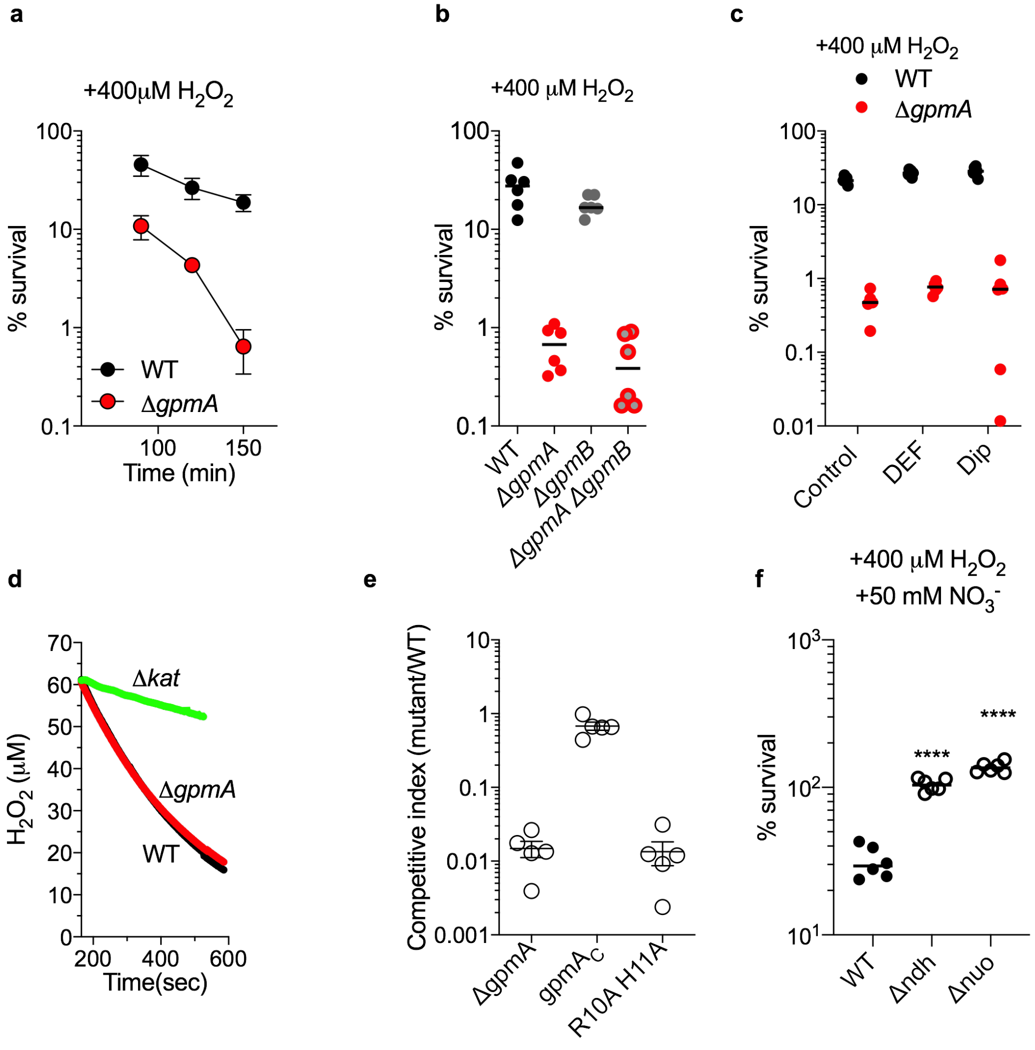

Supplementary Figure 1: Glycolysis contributes to antioxidant defenses of *Salmonella*. (**a**) Susceptibility of Δ*gpmA Salmonella* to 400 μM H_2O_2 as a function of time. Prior to plating, the cells were treated with 200 U/ml of catalase to eliminate persistent oxidative stress. Mean +/- SD; N=5. (**b**) Killing of the indicated *Salmonella* strains 2 h after the addition of 400 μM H_2O_2. Median, N=6. (**c**) Effects of 1 mM of the iron chelators deferoxamine (DEF) or 2,2'dipyridyl (dip) on killing of *Salmonella* by 400 μM H_2O_2. Median, N=6 except control that is N=5. (**d**) Consumption of H_2O_2 was analysed polarographically. A mutant lacking all three catalase genes (*katE*, *katG* and *katN*) was used as control. All data are shown as mean; N=9 except for catalase mutant that was done once. (**e**) Competitive index of the indicated mutants when inoculated i.p. into C57BL/6 mice together with equal numbers of wild-type *Salmonella*. Bacterial burden was quantified in spleen 4 days post inoculation. Mean+/- SEM, N=5. (**f**) Susceptibility of anaerobic *Salmonella* to H_2O_2 in MOPS glucose media supplemented with 50 mM sodium nitrate. Median, N=6. ****, $p < 0.0001$ as determined by one-way ANOVA.

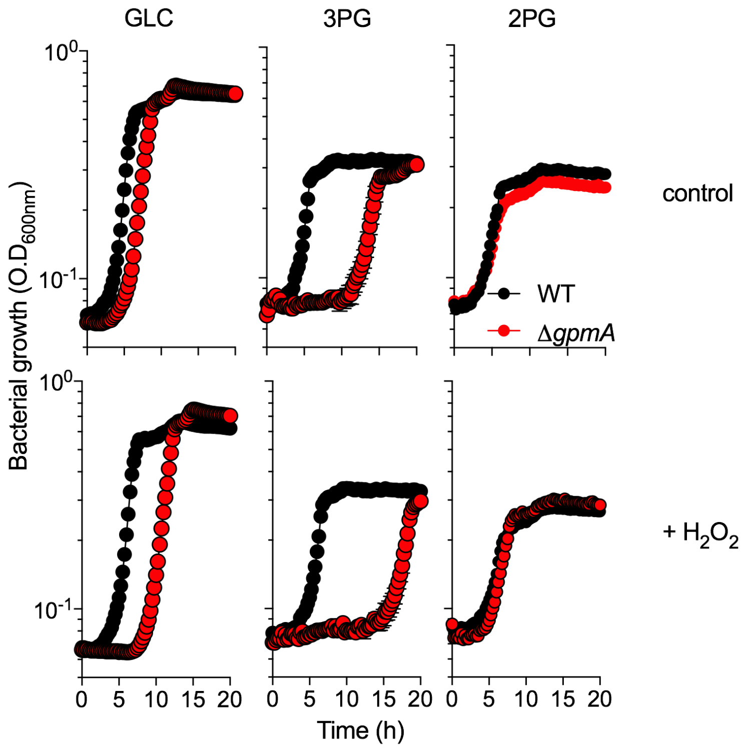

Supplementary Figure 2: Effect of carbon on susceptibility of *Salmonella* to H_2O_2. H_2O_2 toxicity against bacteria grown in the presence of carbon sources that feed above or below phosphoglyceromutase in glycolysis was examined by following bacterial growth in a Bioscreen C plate reader. MOPS minimal media contain 0.4% of either glucose (GLC), 3-phosphoglycerate (3PG), or 2-phosphoglycerate (2PG). Where indicated, H_2O_2 was added at a final concentration of 50 μM at the onset of culture. Representative growth curves are shown of four independent assays containing 4 replicates.

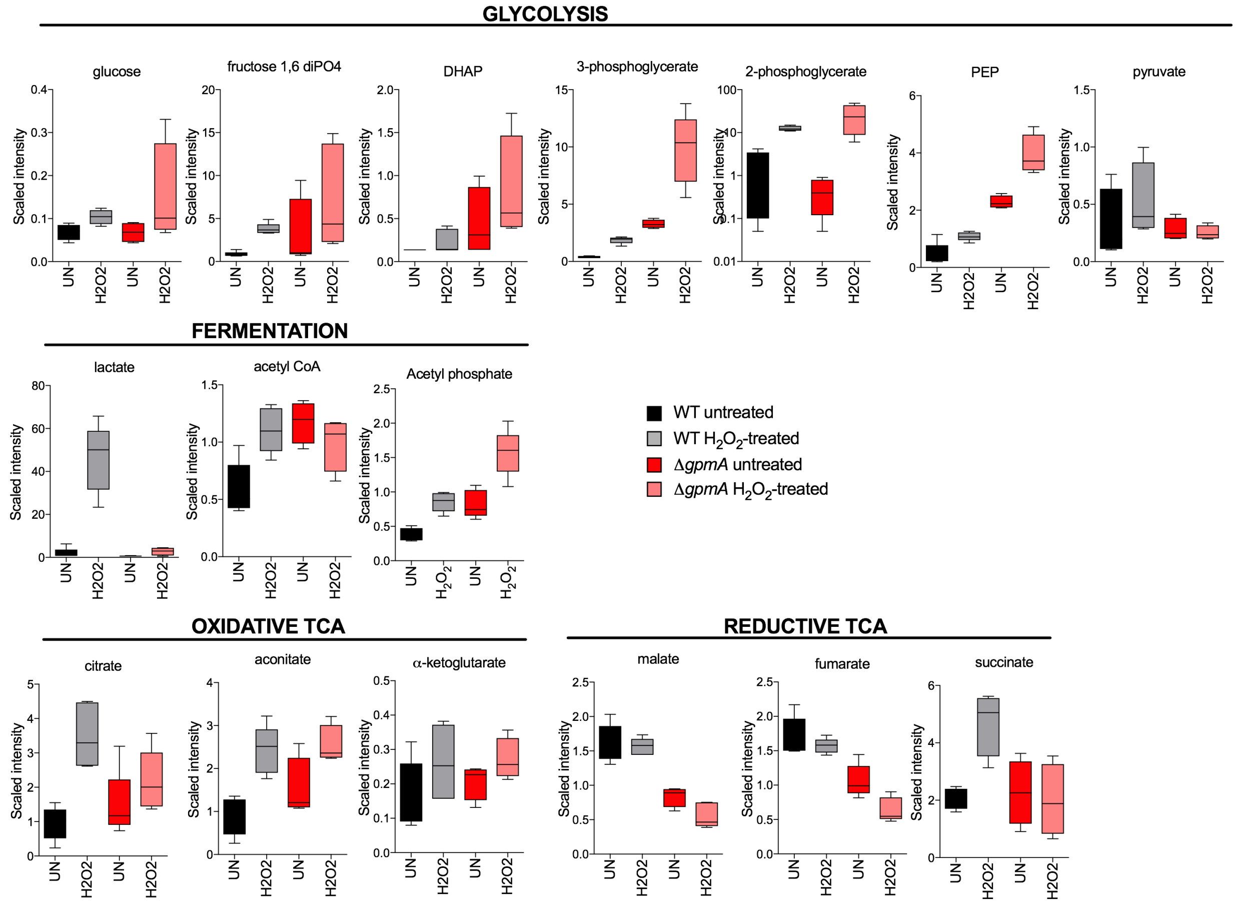

Supplementary Figure 3: Central metabolites in *Salmonella* undergoing oxidative stress. Metabolomics analysis of wild-type (WT) and Δ*gpmA* *Salmonella* grown in MOPS-glucose minimal media. Where indicated, the bacterial cultures were treated with 2.5 mM H_2O_2 for 30 min. The data are from 5 independent experiments. Whiskers in box plots represent minimal to maxima; 25th and 75th percentiles and median are also represented.

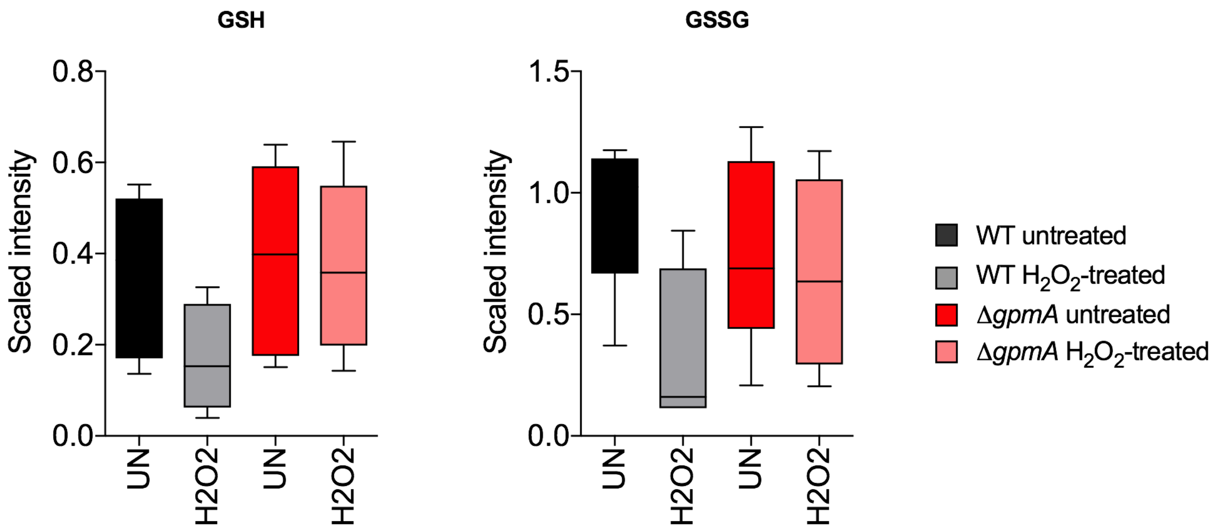

Supplementary Figure 4: Glutathione in *Salmonella* undergoing oxidative stress. Gluthatione (GSH) and oxidized glutathione (GSSG) as determined by metabolomics analysis of wild-type (WT) and Δ*gpmA Salmonella* grown in MOPS-glucose minimal media. Where indicated, the bacterial cultures were treated with 2.5 mM H_2O_2 for 30 min. The data are from 5 independent experiments. Whiskers in box plots represent minimal to maxima; 25th and 75th percentiles and median are also represented.

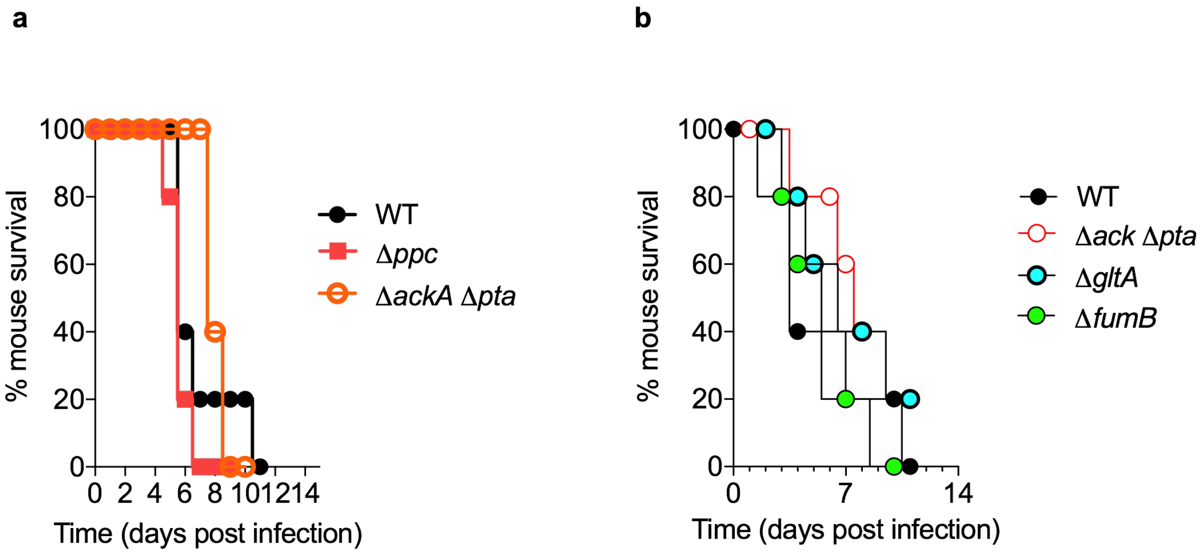

Supplementary Figure 5: Virulence of *Salmonella* in an acute model of systemic infection. C57BL/6 mice were inoculated i.p. with ~200 CFU of either wild-type (WT) or the indicated mutants. N=5.

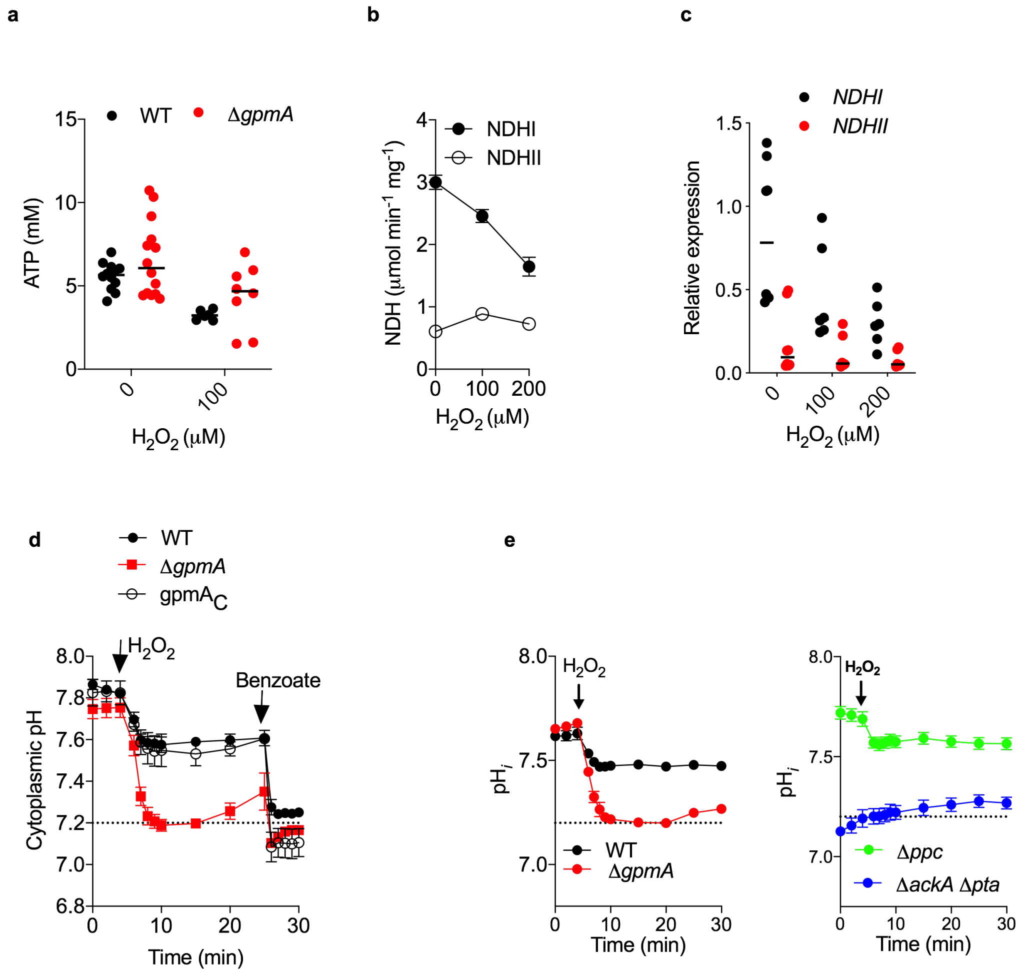

Supplementary Figure 6: Glycolysis preserves ΔpH in *Salmonella* undergoing oxidative stress. (**a**) Intracellular ATP concentrations in wild-type (WT) and Δ*gpmA* *Salmonella* grown to exponential phase in MOPS-glucose minimal media. Some of the samples were treated with 50 μM H_2O_2 for 30 min before ATP determination. Median, N=6 for H_2O_2 and 12 for controls, respectively. (**b**) NADH dehydrogenase enzymatic activity in cell membranes harvested from *Salmonella* 14028s grown to OD_{600} of 0.5 in LB broth for 20 h. Membrane fractions were treated with the indicated H_2O_2 concentrations for 30 min prior to examining for enzymatic activity of proton-coupled and uncoupled NADH dehydrogenases encoded by *nuo* operon and *ndh* gene (NDH-I and NDH-II respectively). Mean ± SD; N=12. (**c**) Expression of *nuoAB* and *ndh* genes in log phase *Salmonella* strain 14028s grown on MOPS-glucose media to OD_{600} of 0.5. Some cultures were treated with the indicated concentrations of H_2O_2 for 30 min before RNA isolation and cDNA synthesis. Expression levels of target genes were quantified by qRT-PCR and normalized to the *rpoD* housekeeping gene. Median, N=6 except N=8 for untreated. (**d, e**) Intracellular pH in the indicated *Salmonella* strains after the addition of H_2O_2 as determined by ratiometric GFP expression of pHluorin. Bacteria were grown to log phase in MOPS glucose media, pH 7.2. Where indicated, the ΔpH was collapsed with benzoate, and some cultures were treated with 400 μM H_2O_2. Mean ± SD; d, N=4; e, N=3.

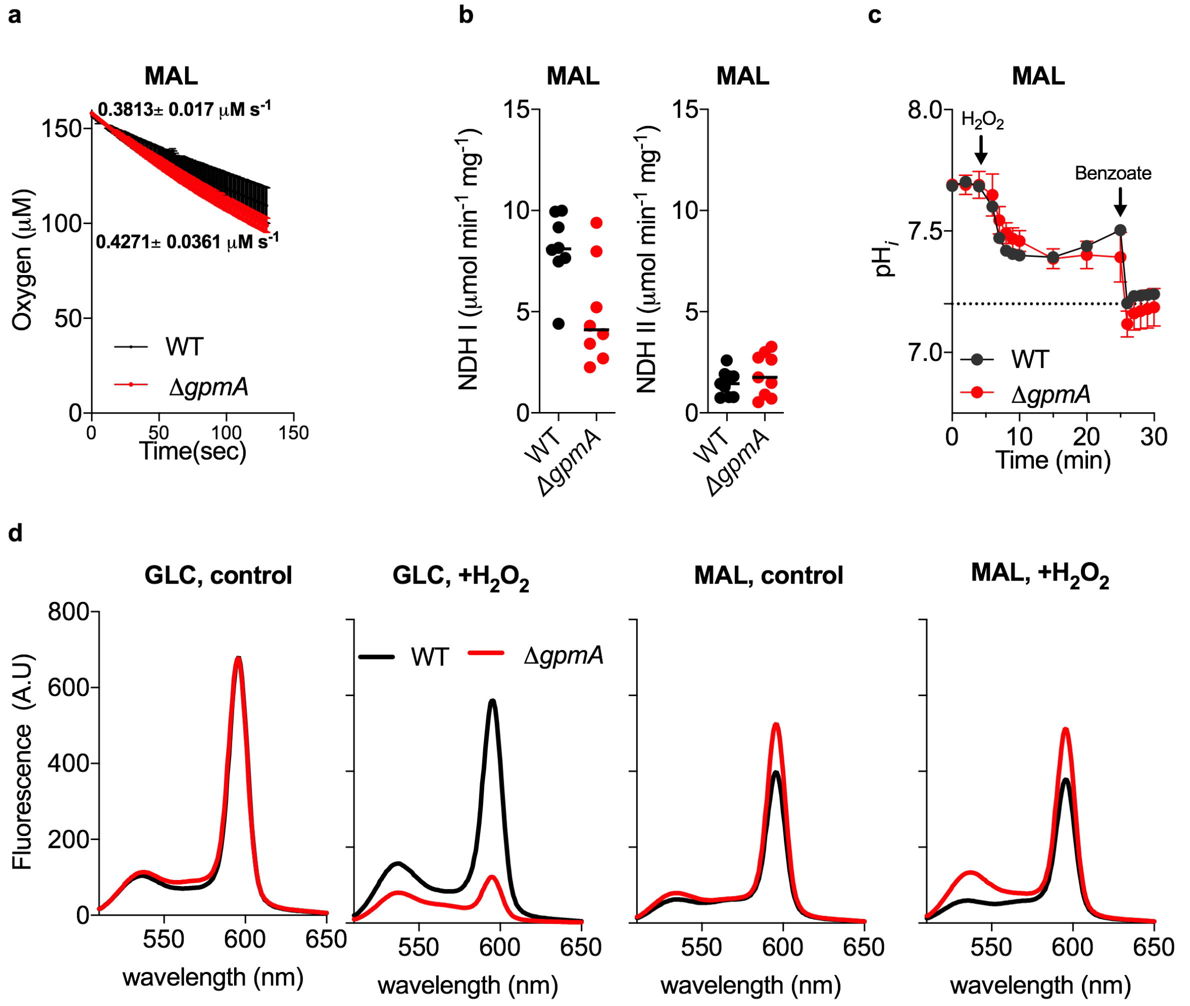

Supplementary Figure 7: Malate reestablishes NDH-I enzymatic activity, preserving PMF in *Salmonella* undergoing oxidative stress. (**a**) Respiratory activity of log phase *Salmonella* grown in MOPS-malate (MAL) media was measured polarographically with an O_2 sensor. Mean ± SD; WT, N=2, Δ*gpmA* N=3. (**b**) Specific activity of NDH-I and NDH-II in the indicated *Salmonella* strains. Enzymatic activity was assayed in bacterial membranes harvested from 50 ml of OD_{600} 0.5 cultures grown in MOPS-malate medium. Median, NDH-I, N=8; NDH-II, N=9. (**c**) Intracellular pH of *Salmonella* was determined by ratiometric GFP expression of the pHluorin construct. Bacterial cells were grown to log phase in MOPS-malate media, pH 7.2. Where indicated, 400 μM H_2O_2 and 40 mM sodium benzoate were added to the cultures. Mean ± SD; WT, N=3; Δ*gmpA*, N=4. (**d**) Proton motive force was measured by recording JC-1-dependent fluorescence. Bacteria were grown in MOPS-glucose (GLC) or –malate (MAL) media to OD_{600} of 0.4. Some samples were treated with 400 μM H_2O_2. The data are representative of 3 independent experiments.

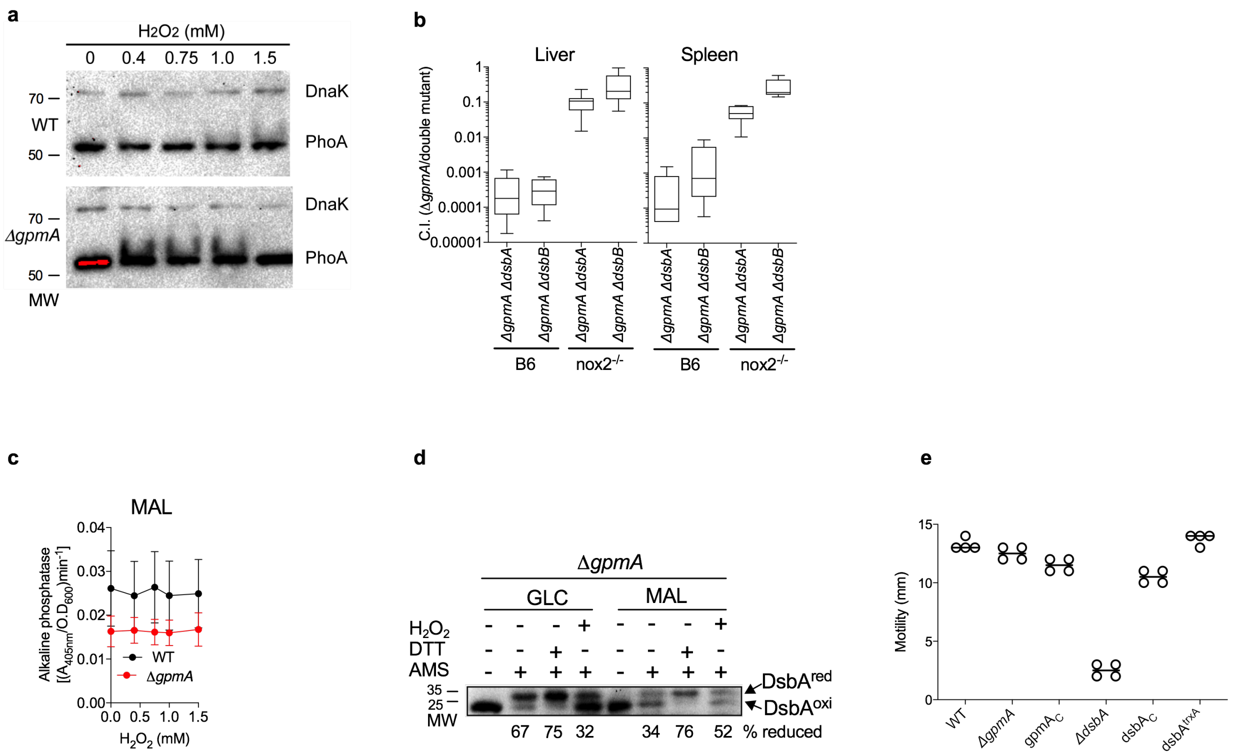

Supplementary Figure 8: Periplasmic function in *Salmonella* undergoing oxidative stress. (**a**) Western blot analysis of *E. coli* PhoA expressed in wild-type (WT) and Δ*gpmA Salmonella* grown to log phase in MOPS-glucose media. Anti-PhoA antibiodies (Catalog number MAB1012) were purchased from Millipore Sigma; anti-DnaK antibodies (MBL International Corporation 50-512-09) were purchased from Fisher Scientific, Hampton, NH. Data representative of 2 independent experiments. (**b**) C57BL/6 (B6) and *nox2*$^{-/-}$ mice were inoculated i.p with of 250 CFU of each of the indicated *Salmonella* strains. The competitive index (CI) was determined in spleens and livers 3 days after infection. Δ*gpmA* Δ*dsbA* in B6, N=5; Δ*gpmA* Δ*dsbA* in *nox2*$^{-/-}$, N=10; Δ*gpmA* Δ*dsbB*, N=8. Whiskers in box plots represent minimal to maxima; 25th and 75th percentiles and median are also represented. (**c**) Alkaline phosphatase enzyme activity monitored in the indicated strains after treatment with increasing concentrations of H_2O_2 for 30 min. The bacteria were grown to log phase in MOPS-malate (MAL) media. Mean ± SD; N=4. (**d**) Western blot of DsbA-3xFLAG expressed in Δ*gpmA Salmonella* after derivatization of thiol groups with the alkylating agent AMS. The bacteria were grown to log phase in MOPS-glucose (GLC) or –MAL media. Where indicated, the bacteria were treated with 10 mM DTT or 1 mM H_2O_2 for 5 min. Data representative of 3 independent experiments. (**e**) Five microliters of *Salmonella* grown overnight in LB broth were spotted on the 0.3% LB agar plates and the capacity to swim into the agar was measured after 3 h of incubation at 37°C. The Δ*gpmA* and Δ*dsbA* mutants were complemented with wild-type alleles (*gpmA*$_C$ and *dsbA*$_C$, respectively). Median, N=4. The blots in S8a and S8d are representative of 3 independent experiments.

Supplementary Table 1: Strains and Plasmids

Strains and Plasmids	Genotype	Source
S. Typhimurium ATCC 14028s	Wild type	ATCC
E. coli DH5α	Cloning strain	Lab stock
AV15133	Δ*gpmA*::FRT	This study
AV18050	Δ*gpmA*::Cm	This study
AV15068	Δ*gpmB*::Km	This study
AV15083	Δ*gpmA*::Cm Δ*gpmB*::km	This study
AV09580	Δ*pfkB*::Km Δ*pfkA*::FRT	[1]
AV16060	Δ*ldhA*::FRT	This study
AV09461	Δ*gltA*::Km	This study
AV09466	Δ*fumB*::Km	This study
AV09474	Δ*ppc*::Km	This study
AV15141	Δ*gpmA*::FRT *put*::*gpmA* R10A H11A::cm	This study
AV19108	Δ*gpmA*::FRT *put*::*gpmA*::cm	This study
AV18202	Δ*gpmA*::FRT Δ*ndh*::Km	This study
AV18201	Δ*gpmA*::FRT Δ*nuo*::Km	This study
AV18115	Δ*ackA*::FRT Δ*pta*::Km	This study
AV19101	Δ*ppc*::FRT Δ*ack-pta*::Cm	This study
AV18143	Δ*fumB*::FRT Δ*ack*-pta::Cm	This study
AV18142	Δ*gltA*::FRT Δ*ack*-pta::Cm	This study
AV0427	Δ*nuo*::FRT	[2]
AV0428	Δ*ndh*::FRT	[2]
AV09372	Δ*dsbA*::Km	This study
AV11037	Δ*dsbB*::Km	This study
AV18026	Δ*gpmA*::FRT Δ*dsbB*::Km	This study
AV18120	Δ*dsbA*::3XFLAG::Km	This study
AV18122	Δ*gpmA*::FRT Δ*dsbA*::3XFLAG::Km	This study
AV18008	Δ*gpmA*::FRT Δ*dsbA*::Km	This study
AV15001	Δ*katE*::FRT Δ*katN*::Km Δ*katG*::Cm	This study
AV19047	14028s pBAD18-*phoA*	This study
AV19048	Δ*gpmA*::FRT pBAD18-*phoA*	This study
AV19106	*put::dsbA*^{trxA}	This study
AV19107	*put::dsbA*^{trxA} pBAD18-*phoA*	This study
AV19102	14028s pHluorin	This study
AV19010	Δ*gpmA*::FRT pHluorin	This study
AV19104	Δ*ppc*::FRT pHluorin	This study
AV19105	Δ*ackA*::FRT *pta*::km FRT pHluorin	This study
AV10109	*E. coli* pSK(+)::*dsbA*::3x-FLAG::Cm	This study
AV19018	*E. coli* pSK(+)::*dsbA* P50GH51P::3x-FLAG::Cm	This study
AV19025	*dsbA* P50GH51P::3x-FLAG::Cm	This study

AV19045	pBAD18::*phoA dsbA* P50GH51P::3x-FLAG::Cm	This study
**Plasmids**		
pCP20	*bla cat* cI857 λP_R *flp* pSC101 oriTS	[3]
pKD13	*bla* FRT::*ahp*::FRT oriR6K	[4]
pKD3	Template vector for FRT-flanked Cm^r cassette, Cm^r Pn^r	[4]
pTP223	P_{lac} *gam bet exo*	[5]
pBAD18	*araC bla rrnB* oriM13 oripBR322	[6]
pBAD18-*phoA*	Overexpression plasmid for *E.coli* PhoA (Pn^r)	This study
pHluorin (pGFPR01)	Ratiometric GFP expression plasmid (Pn^r)	[7]

Supplementary Table 2: Primers

**Gene deletion and point mutation primers**		
gpmA (deletion)	F	5'AGAATTATTATCATTACATATGATTTATAGGAGTGAGAGTTATGAATATCCTCCTTAGTT
	R	5'GCCGGATAAGGCAGTTATACCGCCATCCGGCAAAGGTGAGTGTAGGCTGGAGCTGCTTC
gpmA (*put*)	F	5'-GTCATTGCCAGTGAATTTCTCGCCGATGACGATCAGCGCAAGGTTAAAG
	R	5'- AGTTATACCGCCATCCGGCAAAGGTGAGTGTAGGCTGGAGCTGCTTC
gpmB	F	5'CTCAGTTTTAAAAATTTATCGCAGTATAACGGAAAAAAAACATGGTGTAGGCTGGAGCTGCTTC
	R	5'CTCATCCAATGCAGGGGCGTCCAGATGCGAAACGTCCCCTATTCCGGGGATCCGTCGACC
ldhA	F	5'ATGAAACTCGCCGTCTATAGTACCAAACAGTATGACAAGAGTGTAGGCTGGAGCTGCTTC
	R	5'CCCTGAGCGCAGGGGAGCGACAGGATTAGAACAGCGCGTTATTCCGGGGATCCGTCGACC
ppc	F	5'GGGTGTCTGGGGTCATATGAACGAACAATATTCCGCGTTGGTGTAGGCTGGAGCTGCTTC
	R	5'TTAGCCGGTGTTGCGCATACCGGCAGCGACGCCCGCAATCATTCCGGGGATCCGTCGACC
gltA	F	5'TCCGGCAGTCTTAAGCAATAAGGCGCTAAGGAGACCGTAAGTGTAGGCTGGAGCTGCTTC
	R	5'GTACCGGATGGCGAGGGTTGCGCATCCGGTGTCAAATTCATTCCGGGGATCCGTCGACC
fumB	F	5'AGGTTCTTAATACATTTTCTTACTATTAGGCTGGAAGCACGTGTAGGCTGGAGC
	R	5'AACCGATAACGGGCCCGGGAGAACGCCGGGCCTGCCAGGATTCCGGGGATCCGTCGACC
ackA-pta	F	5'TTAGCCACGTATCATAAATAGGTACTTCCATGGTGTAGGCTGGAGCTGCTTC
	R	5'GGCGTTCACGCCGCCATCCGGCATTAGCTTTTACTGTTACATATGAATATCCTCCTT
dsbA	F	5'CAATTAACGCCAATGTATTAATCGGAGAGAGTTGATCATGGTGTAGGCTGGAGCTGCTTC
	R	5'ATCTTATAAAAACGCCGGTCAGTGACCGGCGTTCTTTTTAATTCCGGGGATCCGTCGACC
dsbB	F	5'VCCGCTAGTGGCGCACGAATTGAATTGGTTTACACTGCGGTTATGAATATCCTCCTTAGTT
	R	5'AACGCTTCGGGCAAAAAAAACGCTCCCGAAGGAGCGTGTGGTGTAGGCTGGAGCTGCTTC
katE	F	5'-CCATTTCAGTGATGAAAGCAGGAGACGAGTTCAATGGTGTAGGCTGGAGCTGCTTC
	R	5'-GAATGCGCCGCATTTTTTGCTGTTTATGCAGGAATCGCGATTCCGGGGATCCGTCGACC
katN	F	5'-GTTGAAGCCAAAAAATAAATAATAAGCAGGAGGCAATATGGTGTAGGCTGGAGCTGCTTC
	R	5'-ACATGACACACGGATTTTACTTGTCGGAACTCACTTGTCATTCCGGGGATCCGTCGACC
katG	F	5'-ATATCGTAACGGTAACACTTTAAAAGGGAGCTGAGATATGGTGTAGGCTGGAGCTGCTTC
	R	5'-TACCGAATAGCAGCCGCTGACGAATTAACCTGTCAGATTACATATGAATATCCTCCTT
gpmA R10A H11A	F	5'-ATGGCTGTAACTAAGCTGGTTCTGGTAGCTGCCGGTGAAAGTCAATGG
	R	5'-CCATTGACTTTCACCGGCAGCTACCAGAACCAGCTTAGTTACAGCCAT
**q-PCR primer**		
nuoAB	F	5'-CAGGAGATCGTAACCGACCC
	R	5'-CACTGCGGTAAAAGAGGTCAC
	P	5'-TACGGCCCCAGTTAACCATGTCA
ndh	F	5'-AAGGCGGTCTGCATACGAAA
	R	5'-CGCCAATCGCATAAATATCAGGA
	P	5'-TTGATACCCGCCGCCCACAC
**Cloning Primers**		
pBAD18-*phoA*	F	5'-CCGGAATTCGTGAAACAAAGCACTATTGCACTGG
	R	5'-CCGTCTAGATTATTTCAGCCCCAGAGCGGCTTTCA
*dsbA*3xFLAG	F	5'-GCTGATACTGTGAAATATTTGGTTGATAAAAAAGACTACAAAGACCATGACGG
	R	5'-CTTATAAAAACGCCGGTCAGTGACCGGCGTTCTTTCATATGAATATCCTCCTTAG
*dsbA*3xFLAG cm	F	5'-AACTCGAGAGCAGACTAAAATTTTGCACGAACC
	R	5'- CCAAGCTTTTACTACTTGTCATCGTCATCCTTGTAGTCGATGTCATGATCTTTATAATCACCGTCATGGTCTTTGTAGTCTTTTTTATCAACCAAATATTTCACAGTATCA
dsbA P50GH51P::3xFlag ::Cm (clone into pSK(+)		5'-CACTTCTTCAAACTGATAACAAGGTCCGCAGTAGAAGGAGAAAAACTCC<br>5'-GGAGTTTTTCTCCTTCTACTGCGGACCTTGTTATCAGTTTGAAGAAGTG
dsbA P50GH51P::3xFlag ::Cm for replacement of		5'-CTTTACAATTAACGCCAATGTATTAATCGGAGAGAGTTGATCATGAAAAAGATTTGGCTG<br>5'-AACATCTTATAAAAACGCCGGTCAGTGACCGGCGTTCTTTGTGTAGGCTGGAGCTGCTTC

native *dsbA* in Salmonella	
pWSK29 *gpmA* F<br>R	5′-CCGCTCGAGCGGATGACGATCAGCGCAAGG<br>5′-CGGGATCCCGTTACTTCGCTTTACCCTGATTC

References:

1 Fitzsimmons, L. *et al.* Zinc-dependent substrate-level phosphorylation powers Salmonella growth under nitrosative stress of the innate host response. *PLoS Pathog* **14**, e1007388, doi:10.1371/journal.ppat.1007388 (2018).

2 Husain, M. *et al.* Nitric oxide evokes an adaptive response to oxidative stress by arresting respiration. *J Biol Chem* **283**, 7682-7689, doi:M708845200 [pii] 10.1074/jbc.M708845200 (2008).

3 Cherepanov, P. P. & Wackernagel, W. Gene disruption in Escherichia coli: TcR and KmR cassettes with the option of Flp-catalyzed excision of the antibiotic-resistance determinant. *Gene* **158**, 9-14, doi:037811199500193A [pii] (1995).

4 Datsenko, K. A. & Wanner, B. L. One-step inactivation of chromosomal genes in Escherichia coli K-12 using PCR products. *Proc Natl Acad Sci U S A* **97**, 6640-6645 (2000).

5 Poteete, A. R. & Fenton, A. C. Lambda red-dependent growth and recombination of phage P22. *Virology* **134**, 161-167 (1984).

6 Guzman, L. M., Belin, D., Carson, M. J. & Beckwith, J. Tight regulation, modulation, and high-level expression by vectors containing the arabinose PBAD promoter. *J Bacteriol* **177**, 4121-4130, doi:10.1128/jb.177.14.4121-4130.1995 (1995).

7 Miesenbock, G., De Angelis, D. A. & Rothman, J. E. Visualizing secretion and synaptic transmission with pH-sensitive green fluorescent proteins. *Nature* **394**, 192-195, doi:10.1038/28190 (1998).
