# Supplementary material for: Glycolytic reprograming in Salmonella counters NOX2-mediated dissipation of ΔpH
Source: Nat Commun. 2020 Apr 14;11:1783. doi: 10.1038/s41467-020-15604-2 (PMC7156505; doi:10.1038/s41467-020-15604-2)
Supplement: Supplementary file 3 — Reporting Summary [file 41467_2020_15604_MOESM3_ESM.pdf]

nature research

Corresponding author(s): Andres Vazquez-Torres
Last updated by author(s): March 4, 2020

Reporting Summary

Nature Research wishes to improve the reproducibility of the work that we publish. This form provides structure for consistency and transparency in reporting. For further information on Nature Research policies, see Authors & Referees and the Editorial Policy Checklist .

Statistics

For all statistical analyses, confirm that the following items are present in the figure legend, table legend, main text, or Methods section.

n/a	Confirmed	
☐	☒	The exact sample size (*n*) for each experimental group/condition, given as a discrete number and unit of measurement
☐	☒	A statement on whether measurements were taken from distinct samples or whether the same sample was measured repeatedly
☐	☒	The statistical test(s) used AND whether they are one- or two-sided<br>*Only common tests should be described solely by name; describe more complex techniques in the Methods section.*
☒	☐	A description of all covariates tested
☒	☐	A description of any assumptions or corrections, such as tests of normality and adjustment for multiple comparisons
☐	☒	A full description of the statistical parameters including central tendency (e.g. means) or other basic estimates (e.g. regression coefficient) AND variation (e.g. standard deviation) or associated estimates of uncertainty (e.g. confidence intervals)
☐	☒	For null hypothesis testing, the test statistic (e.g. *F*, *t*, *r*) with confidence intervals, effect sizes, degrees of freedom and *P* value noted<br>*Give P values as exact values whenever suitable.*
☒	☐	For Bayesian analysis, information on the choice of priors and Markov chain Monte Carlo settings
☒	☐	For hierarchical and complex designs, identification of the appropriate level for tests and full reporting of outcomes
☒	☐	Estimates of effect sizes (e.g. Cohen's *d*, Pearson's *r*), indicating how they were calculated

Our web collection on statistics for biologists contains articles on many of the points above.

Software and code

Policy information about availability of computer code

Data collection	Sequences were obtained using an Illumina HiSeq2500. Barcodes were trimmed from the fastq file, enumerated, and assigned to a known location in the genome, using Perl scripts available upon request.
Data analysis	Statistical analyses were done with GraphPad Prism, Version 8.1.1 (224). Measurement of differences in barcodes used DESeq2 with default parameters (https://bioconductor.org/packages/release/bioc/html/DESeq2.html)

For manuscripts utilizing custom algorithms or software that are central to the research but not yet described in published literature, software must be made available to editors/reviewers. We strongly encourage code deposition in a community repository (e.g. GitHub). See the Nature Research guidelines for submitting code & software for further information.

Data

Policy information about availability of data

All manuscripts must include a data availability statement. This statement should provide the following information, where applicable:

- Accession codes, unique identifiers, or web links for publicly available datasets
- A list of figures that have associated raw data
- A description of any restrictions on data availability

All data generated or analysed during this study are included in this published article and its supplementary information and supplementary data files. Raw data for all figures can be seen in supplementary tables and supplementary excel files. There are no restrictions on data availability.

Field-specific reporting

Please select the one below that is the best fit for your research. If you are not sure, read the appropriate sections before making your selection.

Life sciences study design

All studies must disclose on these points even when the disclosure is negative.

Sample size	Sample size for animal studies was determined taking into account a power of 80%, alpha value of 0.05 and beta value of 0.95.<br>The minimum number of reads was determined as that required to produce at least 7 reads in the input for at least 30,000 different Tn integration sites. 7 because this is the minimum required for a p<0.05 and 30,000 due to cost of acquiring the data.<br>The metabolomics represents the analysis of 5 independent cultures, as recommended by Metabolome, collected on two different days.<br>Sample size for experiments other than metabolomics, Tn seq and mouse experiments were selected based on historical data from our lab that showed statistical power.
Data exclusions	No data were excluded from our analyses.
Replication	Experiments were repeated on 2 independent days at a minimum.<br>Whenever possible, phenotypes were confirmed by two or more independent approaches. For example, several mutants in glycolysis were independently tested; susceptibility of glycolysis mutants to oxidative killing was tested in vitro, in macrophages, and murine models of acute infection; effects of oxidative stress on membrane energetics was monitored by following independently a GFP reporter and JC-1 labeling; and effects of oxidative stress on DpH was monitored on bacterial cultures in vitro and intracellular Salmonella in macrophages. Once a protocol was established, all replicates, independently of their spread, were kept.
Randomization	Mice were assigned at random to any given group. Both sexes were used.
Blinding	Metabolimics was performed blindly by Metabolome, a commercial vendor, which also provided the statistical analysis.<br>Sequencing of Tn libraries was done blindly by a sequencing core at the University of California Irvine.<br>Mouse studies were performed by Lin Liu with bacterial cultures provided unidentified by Sangeeta Chakraborty.<br>Fig. 1b, 1c, 1e, 1f, 2c, 3a-k, 4a, and 4d-h were not done blindly. However, whenever possible, key experiments were done by two individuals.

Reporting for specific materials, systems and methods

We require information from authors about some types of materials, experimental systems and methods used in many studies. Here, indicate whether each material, system or method listed is relevant to your study. If you are not sure if a list item applies to your research, read the appropriate section before selecting a response.

Materials & experimental systems

n/a	Involved in the study	
☐	☒	Antibodies
☒	☐	Eukaryotic cell lines
☒	☐	Palaeontology
☐	☒	Animals and other organisms
☒	☐	Human research participants
☒	☐	Clinical data

Methods

n/a	Involved in the study	
☒	☐	ChIP-seq
☒	☐	Flow cytometry
☒	☐	MRI-based neuroimaging

Antibodies

Antibodies used	anti-FLAG (Catalog number F1804, Sigma-Aldrich, St Louis, MO), anti-PhoA (Catalog number MAB1012), and anti-DnaK ((MBL International Corporaton 50-512-09, Fisher Scientific, Hampton, NH) antibodies
Validation	We validate anti-FLAG antibodies by Western blotting of strains of salmonella expressing the gene of interest tagged with 3XFLAG. Unmarked isogenic strains are used as controls.<br>Anti-PhoA antibodies were tested by probing Salmonella strains expressing empty pBAD or pBAD-phoA plasmids.<br>Anti-DnaK antibodies were confirmed by comparing expression between wild-type and DdnaK Salmonella strains.

Animals and other organisms

Policy information about studies involving animals; ARRIVE guidelines recommended for reporting animal research

Laboratory animals	mice, C57BL/6, nox2-/-, males and females, 6-8 week old. In house bredding from stocks obtained from Jackson labs. The genotype of nox2-/- mice was confirmed by genetically by PCR. Mice in the colony are periodically phenotyped by following the ability of periodate-elicited macrophages to sustain lucigenin-dependent chemiluminescence. Macrophages isolated from congenic C57BL/6 mice serve as controls.
Wild animals	No wild animals were used in these studies

Field-collected samples	No field-collected samples were used in these studies
Ethics oversight	University of Colorado School of Medicine IACUC

Note that full information on the approval of the study protocol must also be provided in the manuscript.
